# Molecular and Functional Analysis of the Stearoyl-CoA Desaturase (SCD) Gene in Buffalo: Implications for Milk Fat Synthesis

**DOI:** 10.3390/ani14223191

**Published:** 2024-11-07

**Authors:** Wenbin Dao, Xinyang Fan, Jianping Liang, Tao Chen, Zaoshang Chang, Yongyun Zhang, Yongwang Miao

**Affiliations:** 1 College of Animal Science and Technology, Yunnan Agricultural University, Kunming 650201, China; dwbin666@126.com (W.D.); xinyangfan1@ynau.edu.cn (X.F.); xxscmyt1@126.com (Z.C.); 2 Institute of Animal Genetics and Breeding, Yunnan Agricultural University, Kunming 650201, China; zyypang@126.com; 3 Science and Technology Innovation Center of Dehong Prefecture, Mangshi 678400, China; yndhkjjrck@126.com; 4 Mangshi Animal Husbandry Station, Mangshi 678400, China; ynlxxmz@126.com; 5 College of Veterinary Medicine, Yunnan Agricultural University, Kunming 650201, China

**Keywords:** buffalo *SCD* gene, molecular characterization and function, milk fat synthesis, association analysis

## Abstract

The SCD is a rate-limiting enzyme that catalyzes the synthesis of monounsaturated fatty acids (MUFAs) in dairy cows; however, its role in the mammary gland of buffalo is not well understood. In this study, we isolated and characterized the complete coding sequence (CDS) of the buffalo *SCD* gene from mammary gland tissue and investigated its effects on milk fat synthesis using bioinformatics analyses, tissue differential expression detection, and cellular functional experiments. The cloned *SCD* gene has a CDS length of 1080 bp, encoding a protein of 359 amino acids. This protein is hydrophilic, lacks a signal peptide, and contains four transmembrane domains, including 10 conserved motifs and a Delta9-FADS domain, characteristic of the fatty acid desaturase family involved in unsaturated fatty acid biosynthesis within the endoplasmic reticulum. Molecular characterization revealed that the physicochemical properties, conserved domains, structures, and functions of buffalo SCD are highly similar to those in other Bovidae species. Among the tissues analyzed, *SCD* expression was highest in the mammary gland during lactation and in the cerebellum during dry-off period. Notably, *SCD* expression in the mammary gland was significantly higher during lactation compared to the dry-off period. Subcellular localization experiments confirmed that SCD functions in the endoplasmic reticulum of buffalo mammary epithelial cells (BuMECs). Functional overexpression and interference experiments in BuMECs demonstrated that SCD promotes milk fat synthesis by affecting the expression of lipid synthesis-related genes such as *ACACA*, *FASN*, and *DGAT1*, as well as milk fat regulatory genes like *SREBFs* and *PPARG*, thereby influencing intracellular triglyceride (TAG) content. Additionally, 18 single-nucleotide polymorphisms (SNPs) were identified in the buffalo *SCD* gene, with a specific SNP at c.-605, showing potential as molecular markers for improving milk production traits. These findings highlight that the *SCD* gene is a key gene in buffalo milk fat synthesis, involved in the de novo synthesis of milk fatty acids.

## Simple Summary

SCD plays a crucial role in the synthesis of monounsaturated fatty acids in dairy cows; however, its role in the mammary gland of buffalo is not well understood. In this study, the buffalo *SCD* gene CDS was isolated and characterized, and its molecular characterization, tissue expression, functions, and polymorphisms were analyzed. The results showed that the molecular characterization of buffalo SCD was similar to that of other Bovidae species, and its expression level in the mammary gland during lactation was significantly higher than during dry-off period period. Functional experiments revealed that SCD plays an important role in the endoplasmic reticulum of BuMECs for fatty acid synthesis in milk. Additionally, we found that c.-605A>C in the *SCD* gene was associated with milk yield in buffalo. These findings provide new perspectives for comprehending the mechanism of milk fat synthesis in buffalo and provide the basis for the selection of buffalo lactation traits.

## 1. Introduction

SCD, also known as delta-9 desaturase, fatty acid desaturase 5 (FADS5), stearoyl-CoA desaturase 1 (SCD1), or stearoyl-CoA desaturase opposite strand (SCDOS) (as referenced in the GeneCards database: https://www.genecards.org/, accessed on 28 June 2024), belongs to the family of fatty acid desaturases. The *SCD* gene was first identified in the liver of rats [1]. The enzyme it encodes, SCD, resides in the endoplasmic reticulum, where it catalyzes the formation of a cis-double bond at the delta-9 position of substrates, making it the rate-limiting enzyme in the de novo synthesis of MUFA [2,3]. In humans, *SCD* is expressed in adipose tissue, heart, brain, liver, and lungs, with the highest expression observed in adipose tissue [4]. In cattle, the highest expression of the *SCD* gene is also found in adipose tissue, followed by the heart, brain, and muscle [5]. In cattle mammary glands, *SCD* exhibits higher catalytic activity towards the substrate of long-chain saturated fatty acids than other substrates, and a total of 14 SNPs have been identified in the promoter region, intron 1, exon 5, and 3’UTR of this gene, in which the milk from cows carrying the rs523411937 CT genotype had significantly lower levels of C15:0 than that from individuals with the CC genotype [6]. Furthermore, at the g.10329C>T locus of the *SCD* gene in Angus cattle, the intermuscular monounsaturated fatty acid content of individuals carrying the C allele was significantly higher than that of individuals carrying the T allele [7].

To date, the *SCD* gene has been isolated in several domesticated bovids, including sheep, goats, cattle, and buffalo. The cattle *SCD* gene is located on BAT26 and spans 17,088 bp, comprising six exons and five introns, with an mRNA length of 5287 bp and a CDS of 1080 bp encoding 359 amino acids [8,9]. In buffalo, the *SCD* gene is located on BBU23, with a total length of 15,166 bp, which also includes six exons and five introns, with an mRNA length of 5082 bp and a CDS length of 1080 bp (https://www.ncbi.nlm.nih.gov/, accessed on 28 June 2024). In goat, the *SCD* gene is located on CHI26, with a full gene length of 1138 bp and a CDS length of 1080 bp (https://www.ncbi.nlm.nih.gov/, accessed on 28 June 2024). SCD plays a crucial role in various physiological processes related to lipid metabolism [10,11], and it has the potential to be a candidate gene for improving milk and meat quality. However, the specific role and mechanisms of the *SCD* gene in milk fat synthesis in buffalo remain unclear.

The buffalo (*Bubalus bubalis*) is an important source of meat, milk, and draft power in many tropical and subtropical countries [12]. Domestic buffalo are categorized into river and swamp types, with the river type primarily used for milk production and the swamp type mainly for draft purposes. Compared to Holstein cow milk, buffalo milk contains higher levels of total solids, fat, and protein [13]. The fatty acid composition of buffalo milk is approximately 70.49% saturated fatty acids (SFAs), 25.95% monounsaturated fatty acids (MUFAs), and 3.54% polyunsaturated fatty acids (PUFAs), with palmitic acid (C16:0), oleic acid (C18:1), myristic acid (C14:0), and stearic acid (C18:0) as the main fatty acids [14]. In dairy cow milk, SFAs, MUFAs, and PUFAs account for approximately 70%, 25%, and 2.3%, respectively, with similar main fatty acids [15]. Due to the nutritional advantages of buffalo milk, buffalo have become the second-largest source of milk worldwide [16]. Investigating the functions and molecular mechanisms of lactation-related genes in buffalo will provide a foundation for breeding and nutritional regulation. In this study, the *SCD* gene of buffalo was isolated and identified, and the gene was further studied using bioinformatics analysis, tissue differential expression detection, cell function testing, and association analysis between variation and traits, in order to reveal the role and mechanism of the *SCD* gene in the synthesis of buffalo milk fat and provide a basis for the breeding of lactation traits in buffalo.

## 2. Materials and Methods

### 2.1. Animal Sources and Sample Collection

Sample collection for gene cloning and tissue differential expression analysis involved six healthy adult female Binglangjiang buffalo, three in lactation (about 60 d postpartum) and three in dry-off (about 60 d before parturition), which were approximately 5 years old and were selected from a Binglangjiang Buffalo Farm in Tengchong, Yunnan Province, China. The buffalo, all unrelated by blood and maintained under identical conditions, were subjected to tissue sample collection post-slaughter. The buffalo slaughtering was carried out in accordance with the Operating Procedures of Cattle Slaughtering (GB/T 19477-2004) [17]. Tissue samples from the heart, liver, spleen, lungs, kidneys, muscles, brain, cerebellum, mammary gland, pituitary gland, small intestine, rumen, skin, and ovary were immediately frozen in liquid nitrogen and transported to the laboratory for RNA extraction.

Blood samples for population variation detection were collected from 184 Binglangjiang buffalo (river type) and 40 Dehong buffalo (swamp type) from farms in Tengchong and Mangshi, Yunnan Province, China, respectively. All buffalo were adult and unrelated by blood. About 5 mL of blood were drawn from the jugular vein, placed in EDTA-anticoagulated tubes, and transported to the lab at a low temperature for DNA extraction.

### 2.2. RNA Extraction, cDNA Synthesis, and DNA Extraction

RNA extraction from tissue samples was performed using Trizol reagent (Invitrogen, USA). RNA concentration and purity assessment utilized a NanoDrop 2000 UV spectrophotometer (Thermo Fisher Scientific, Waltham, MA, USA), with RNA integrity confirmation via 1% agarose gel electrophoresis. The synthesis of cDNA employed the PrimeScript™ RT reagent Kit (TaKaRa, Dalian, China), with dilution to 100 ng/µL and storage at −20 °C for subsequent use.

Genomic DNA extraction from blood samples was conducted using the phenol/chloroform method [18], with concentration and purity measurement performed by a NanoDrop 2000 UV spectrophotometer (Thermo Fisher Scientific, Waltham, MA, USA). The extracted DNA was diluted to 100 ng/µL in TE buffer (Servicebio, Wuhan, China) and stored at 4 °C.

### 2.3. Isolation and Identification of Buffalo SCD

Primers for isolating the buffalo *SCD* CDS were designed using Primer Premier 5 software [19] based on the buffalo mRNA sequence (NM_001290915 [20]) (Table 1). The PCR reaction (20 μL) included 1 μL of mammary cDNA template, 1 μL of each primer, 12 μL of 2 × PCR Master Mix (CWBIO, Beijing, China), and 5 μL of ddH_2_O. The reaction involved pre-denaturation at 94 °C for 5 min, followed by 34 cycles of denaturation at 94 °C for 30 s, annealing at 59.7 °C for 30 s, and extension at 72 °C for 65 s, with a final extension at 72 °C for 8 min and termination at 4 °C. PCR products were detected via 1% agarose gel electrophoresis. The target band was excised and purified using the TIANgel kit (TIANGEN, Beijing, China). The purified products were ligated into the pMD-18T vector (TaKaRa, Dalian, China) and cloned. Thirty monoclonal colonies were selected for bidirectional sequencing.

Sequence proofreading employed SeqMan within the Lasergene software package version 7.1 (DNAStar, Inc., Madison, WI, USA), with Open Reading Frame (ORF) identification conducted using the ORF Finder program (https://www.ncbi.nlm.nih.gov/orffinder/, last accessed on 5 May 2024). Verification of the *SCD* gene sequence was conducted through a homology search using the NCBI BLAST program (https://blast.ncbi.nlm.nih.gov/Blast.cgi, last accessed on 5 May 2024).

### 2.4. Molecular Characterization, Structure, and Function Analysis

To elucidate the sequence characterization of the buffalo *SCD* gene, the *SCD* gene and its encoded protein sequences of 12 species were retrieved from the NCBI database for comparative analysis (Table 2). Gene structure determination employed GTF files from the NCBI database (https://www.ncbi.nlm.nih.gov/datasets/, last accessed on 28 June 2024), which were processed using TBtools [21] and visualized using the Gene Structure Display Server 2.0 [22]. Analysis of SCD consistency and divergence was conducted using MegaAlign within the Lasergene 7 software package (DNAStar, Inc., Madison, WI, USA). Phylogenetic analysis of the SCD protein utilized the maximum likelihood method (JTT+G matrix model) in MEGA7 [23]. Identification of conserved motifs was performed using the MEME website [24], and conserved domains were analyzed using the NCBI Batch Web Search tool (https://www.ncbi.nlm.nih.gov/Structure/bwrpsb/bwrpsb.cgi, last accessed on 28 June 2024), with results visualized using TBtools. Prediction of the physicochemical properties of the SCD protein was carried out using ProtParam [25]. Prediction of secondary and three-dimensional structures was carried out using SOPMA [26] and SWISS-MODEL [27], respectively. Prediction of SCD amino acid modification sites was conducted using Prosite Scan [28]. The hydrophilicity, signal peptide, and transmembrane domain of SCD were predicted using the online software ProtScale 1.0 [25], the SignalP5.0 server [29], and the TMHMM 2.0 server [30], respectively. Protein interaction prediction employed STRING [31], and subcellular localization prediction was carried out using ProtComp (http://www.softberry.com/, accessed on 28 June 2024). Functional and biological process analysis was performed using InterProScan [32] and the DAVID [33].

### 2.5. Tissue Differential Expression Analysis

*SCD* gene expression across various tissues was quantified using an iQ5 Real-Time PCR instrument (Bio-Rad, Hercules, CA, USA). Primers were designed with Primer Premier 5, as shown in Table 1. RT-qPCR was performed using SYBR Green Real-Time PCR Mix (Takara, Dalian, China) according to the manufacturer’s instructions. The β-actin (*ACTB*) gene served as the internal reference for normalizing gene expression. Amplification efficiency was assessed using the LinRegPCR program, with all experiments performed in triplicate. Data analysis and visualization were conducted using GraphPad Prism 5 (GraphPad Software Inc., La Jolla, CA, USA). RT-qPCR data were analyzed using the 2^−∆∆Ct^ method. Statistical analysis was performed using one-way ANOVA and Tukey’s method, with a significance threshold set at *p* < 0.05.

### 2.6. Functional Analyses at the Cellular Level

#### 2.6.1. Cell Culture

BuMECs previously isolated and characterized in our laboratory [34] were used in this study. The BuMECs were cultured in DMEM (Gibco, New York, NY, USA) supplemented with 5 μg/mL hydrocortisone (Sigma, St. Louis, MO, USA), 5 μg/mL insulin (Sigma, St. Louis, MO, USA), 1 μg/mL epidermal growth factor (Sigma, St. Louis, MO, USA), 2% penicillin/streptomycin (Gibco, USA), and 10% fetal bovine serum (Gibco, USA). Incubation occurred at 37 °C in a humidified atmosphere containing 5% CO_2_, with medium changes every 48 h. Lactation induction was performed with 3 µg/mL prolactin (Sigma, St. Louis, MO, USA) prior to the experiment [35].

#### 2.6.2. Construction of Overexpression Recombinant Vectors

Buffalo mammary tissue cDNA was used to amplify the *SCD* gene CDS without a stop codon (primer details shown in Table 1). PCR systems and procedures are described in Section 2.3. The amplified product was ligated into the pMD18-T vector (TaKaRa, Dalian, China) for cloning. Following dual enzymatic digestion, it was ligated into the pEGFP-N1 vector, and the recombinant vector pEGFP-N1-*SCD* was constructed. All vector constructs were verified by restriction digestion and sequencing.

#### 2.6.3. *SCD* Overexpression in BuMECs

Culturing of BuMECs in six-well plates proceeded until cell confluence reached 70–80%. The transfection of the pEGFP-N1-*SCD* recombinant vector into the BuMECs was executed following the instructions provided with the TransIntro^®^ EL Transfection Reagent (TransGen Biotech, Beijing, China). The transfection of pEGFP-N1 served as a negative control. Forty-eight hours post-transfection, the BuMECs were collected for subsequent RT-qPCR analysis.

#### 2.6.4. *SCD* Interference in BuMECs

The design and synthesis of two siRNAs targeting the *SCD* CDS (siRNA1-*SCD* and siRNA2-*SCD*), along with a non-specific control (NC) siRNA, were carried out by Shanghai Bioengineering Co., with details provided in Table 1. The transfection of the siRNA with the highest interference efficiency (100 nM) into BuMECs was performed when the cultured cells reached 70–80% confluence in a six-well plate, following the instructions for the TransIntro^®^ EL Transfection Reagent (TransGen Biotech, Beijing, China). Forty-eight hours post-transfection, the BuMECs were harvested for RT-qPCR analysis.

#### 2.6.5. qPCR Analysis of Genes Related to Milk Fat Synthesis

RT-qPCR was used to detect the expression of genes related to milk fat synthesis in BuMECs, with *ACTB* as an internal reference. Primer details are provided in Table 1, and all target gene amplification products were verified by sequencing. RT-qPCR data were analyzed using the 2^−∆∆Ct^ method. All data are expressed as means ± SEM. Statistical analysis was performed using Student’s *t*-test, with significance thresholds of *p* < 0.05 (significant) and *p* < 0.01 (highly significant).

### 2.7. Subcellular Localization of SCD in BuMECs

After transfecting the BuMECs after 48 h with the constructed pEGFP-N1-*SCD* recombinant vector, mitochondria and nuclei were stained with 200 nM Mito-Tracker Red CMXRos (Beyotime, Shanghai, China) and Hoechst 33342 (Beyotime, Shanghai, China), respectively. Observation of SCD protein localization was carried out using a confocal laser scanning microscope (OLYMPUS, Tokyo, Japan).

### 2.8. Polymorphism Detection and Association Analysis

Primers were designed using Primer Premier 5 to detect polymorphisms in the *SCD* CDS and promoter region (−565–−41, recorded with the first nucleotide of the start codon in the buffalo *SCD* gene coding sequence as +1) using sequences FN395259 and NC_059179 as templates (Table 1). The PCR reaction system (total volume 25 µL) consisted of 1 µL of DNA template (100 ng/µL), 2.5 µL of PCR buffer (Mg^2+^ Plus), 0.4 µL of each primer (10 μM), 2.0 µL of dNTP (2.5 mM), 0.3 µL of rTaq enzyme (5 U/µL), and 18.4 µL of ddH_2_O. The PCR protocol included an initial denaturation at 95 °C for 5 min, followed by 34 cycles of denaturation at 95 °C for 40 s, annealing at 50.8–61 °C (Table 1) for 40 s, and extension at 72 °C for 60 s, with a final extension at 72 °C for 5 min and termination at 4 °C. Detection of PCR products was achieved by 1% agarose gel electrophoresis, followed by bidirectional sequencing at Shanghai Sangong Biological Engineering Co.

Sequence analysis involved comparison, mutation site identification, and output using SeqMan in the Lasergene 7 software package (DNAStar, Inc., Madison, WI, USA). Estimations of genotype and gene frequencies, along with Hardy–Weinberg equilibrium tests, were conducted using PopGen 32 software [36]. Prediction of the impact of amino acid substitutions on protein function was performed using the PANTHER program (http://www.pantherdb.org/, last accessed on 28 June 2024).

The association analysis between SNP genotypes within the *SCD* gene CDS and promoter region and observed lactation traits in buffalo was conducted using the following model: Y_ijm_ = μ + G_i_ + e_ijm_; where Y_ijm_ represents the observed value of the lactation trait, *μ* is the population mean, G_i_ is the effect of the i-type of the SNP locus, and e_ijm_ is the random error. Statistical analysis was conducted using the General Linear Model (GLM) procedure in SAS 9.3 software (SAS Inc., Cary, NC, USA). Results were expressed as least square means ± standard error, with statistical significance determined at *p* < 0.05 (significant) and *p* < 0.01 (highly significant).

## 3. Results

### 3.1. Isolation and Identification of SCD Sequence

**Figure 1:**
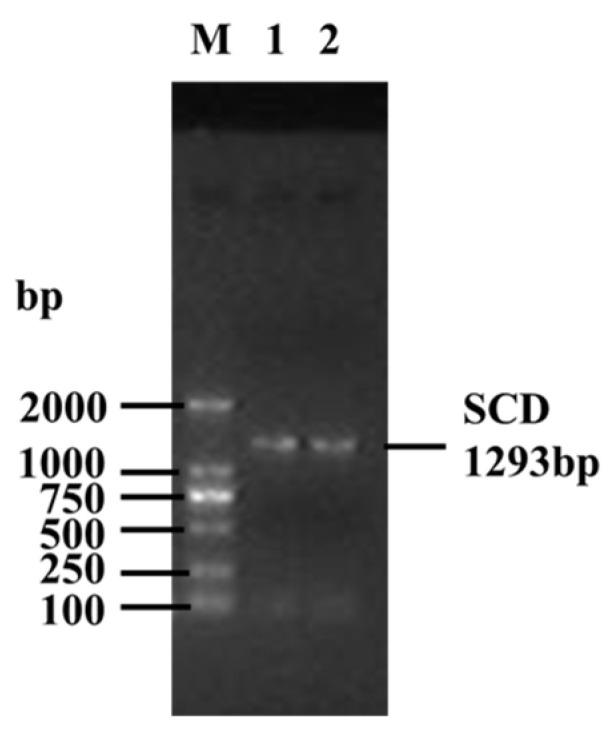
RT-PCR gel electrophoresis of buffalo *SCD* gene CDS. Lane M shows the DL2000 DNA marker, and lanes 1 and 2 show the PCR products of buffalo *SCD* gene CDS.

**Figure 2:**
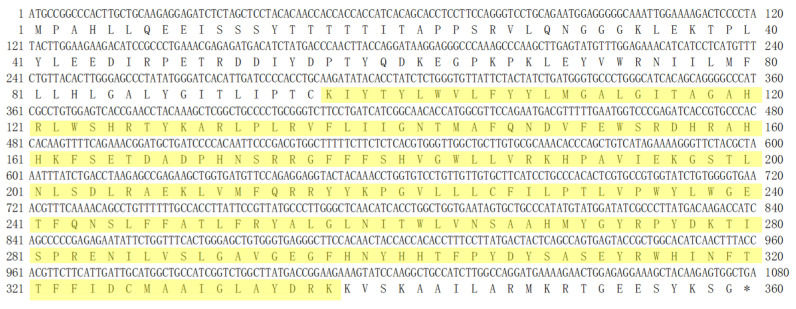
The CDS of buffalo *SCD* gene and its corresponding amino acid sequence. The yellow-highlighted region represents the conserved domain of the buffalo SCD protein (Delta9-FADS-like, amino acids (AAs) 97-337), and the stop codon is denoted by “*”.

The PCR product had a length of 1293 bp (Figure 1), and sequencing of the positive monoclonal bacterial liquid revealed only a single transcript. The ORF Finder program identified the CDS as 1080 bp. A homology search in the NCBI database revealed that this CDS shares over 98.15% similarity with the *SCD* CDS of other Bovidae species. Consequently, the sequence was confirmed as the buffalo *SCD* sequence and has been submitted to the NCBI database under accession number PP950448. The nucleotide composition of the *SCD* gene CDS is as follows: A accounts for 24.35%, T for 24.54%, C for 27.41%, and G for 23.70%. The G+C content is 51.11%, and the A + T content is 48.89% (Figure 2).

### 3.2. Structure of the Transcript Region

**Figure 3:**
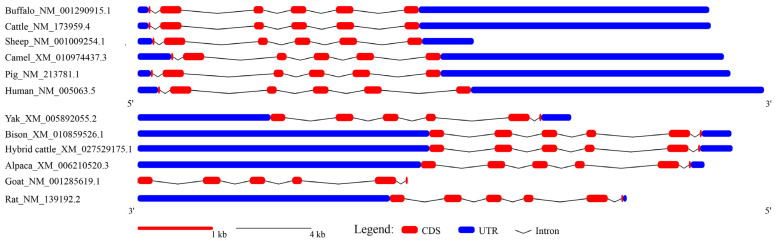
Structure of the transcribed region of the *SCD* gene in buffalo and other mammals.

To investigate the structural characteristics of the buffalo *SCD* transcript region, we aligned the buffalo *SCD* sequence with homologous sequences from 11 other mammals (Figure 3). To date, only a single *SCD* mRNA sequence has been reported for each Bovidae species, including buffalo, cattle, yak, hybrid cattle (zebu × cattle), bison, goat, and sheep, all of which consist of six exons, with a CDS length of 1080 bp (Table 2). With the exception of exon 1 and exon 6, the exon lengths were conserved across Bovidae species (Table 2). Additionally, the UTR lengths varied between buffalo and most other species (Table 2, Figure 3). Notably, the *SCD* gene in buffalo, cattle, sheep, camel, pig, and human are located on the sense strand, whereas in bison, yak, hybrid cattle, alpaca, goat, and rat, they are found on the antisense strand (Figure 3). Overall, the buffalo *SCD* gene has a similar transcript structure to other mammals, particularly in its coding region (Figure 3).

### 3.3. Physicochemical Characteristics and Functional Modification Sites of Buffalo SCD

To reveal the physicochemical characteristics of buffalo SCD, the protein sequence encoded by the buffalo *SCD* gene was analyzed using bioinformatics and compared with SCD proteins from other Bovidae species. The results showed that the physicochemical characteristics of buffalo SCD are similar to those of other Bovidae species (Table 3). The instability index and overall average hydrophilicity of the buffalo SCD protein were 47.21 and −0.193, respectively, indicating that the protein is unstable and hydrophilic. The buffalo SCD protein lacks a signal peptide but contains four transmembrane domains (TMhelix1: AA71–93, TMhelix2: AA98–120, TMhelix3: AA221–238, TMhelix4: AA251–273). Both buffalo’s and other Bovidae species’ SCD proteins have six potential functional modification sites: casein kinase II phosphorylation sites (AA58–61, 164–167, 166–169, 309–312, 351–354), protein kinase C phosphorylation sites (AA95–97, 124–126, 127–129, 173–175, 355–357), N–myristoylation sites (AA85–90, 114–119, 141–146, 197–202, 257–262), N–glycosylation sites (AA201–204, 259–262, 318–321), cAMP– and cGMP–dependent protein kinase phosphorylation sites (AA337–340), and tyrosine kinase phosphorylation site 2 (AA349–356) (Table 4).

### 3.4. Sequence Identity and Phylogeny, Motif Composition, and Conserved Structural Domain Analysis

**Figure 4:**
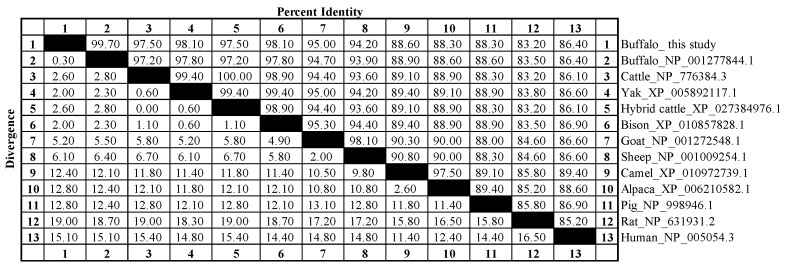
Sequence conservation and divergence of SCD proteins between buffalo and 11 other mammalian species. The values above the diagonal indicate consistency, and those below represent divergence.

**Figure 5:**
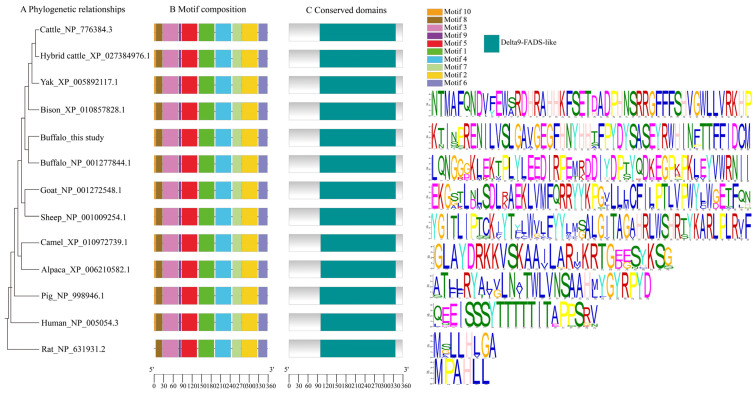
Phylogenetic relationships, motif composition, and conserved structural domains. (**A**) Phylogenetic relationships constructed on the basis of 12 mammalian SCD sequences; (**B**) motif composition of SCD for each species; (**C**) conserved structural domains of SCD across species. Colored boxes indicate different conserved motifs and conserved structural domains in SCD.

Sequence analysis showed that the amino acid sequences of buffalo SCD identified closely with those of other Bovidae species, which ranged from 94.2% to 99.7%. Among them, the SCD of buffalo in this study showed 97.5% similarity with that of cattle, and 98.1% similarity with that of yak and bison (Figure 4). Phylogenetic analysis based on SCD amino acid sequences showed that buffalo and other Bovidae species clustered in a single branch, suggesting that SCD sequences among Bovidae species differ little and are functionally similar (Figure 5A). Further analysis showed that both buffalo and other mammals contain ten similar motifs (except for rat, which is missing motif 10, Figure 5B) and a conserved structural domain of Delta9-FADS (Figure 5C), which is part of the fatty acid desaturase family. The Delta9-FADS-like domain contains motif 1, motif 2, motif 4, and motif 7 (Figure 5).

### 3.5. Secondary and Tertiary Structure of Buffalo SCD

**Figure 6:**
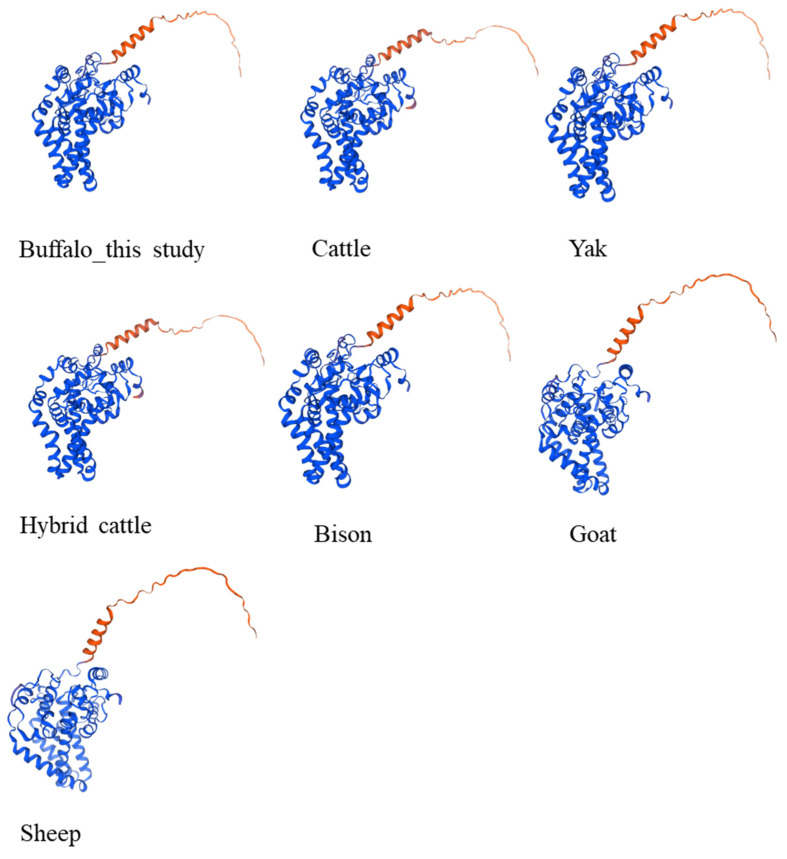
The 3D structure of the SCD proteins in buffalo and other Bovidae species.

The predicted secondary structure of the buffalo SCD protein closely resembles that of SCD proteins from other Bovidae species (Table 5). The random coil content of buffalo SCD differs from that of bison by only 0.56%. The α-helix content in buffalo is identical to that of goats and sheep, with all having a value of 37.05%. The β-turn content in buffalo varies by only 0.27% compared to bison and yak, while the extended strand content differs from bison by only 0.28%. The three-dimensional (3D) structure of the buffalo SCD protein, constructed using homology modeling, is shown in Figure 6. The template for this model is A0A1S3F9W9.1.A (Ord’s kangaroo rat), with a sequence identity of 81.51% and 100% coverage. The 3D structure of the buffalo SCD protein is highly similar to that of other Bovidae species (Figure 6).

### 3.6. Protein Interactions, Biological Processes, and Molecular Functions

The prediction results indicate that buffalo SCD protein is primarily involved in the biological process (BP) of the monounsaturated fatty acid biosynthetic process (GO:1903966, https://www.ebi.ac.uk/QuickGO/, accessed on 28 June 2024). Its interacting proteins include acetyl-CoA carboxylase alpha (ACACA), fatty acid synthase (FASN), elovl fatty acid elongase 6 (ELOVL6), stearoyl-CoA desaturase 5 (SCD5), 3-hydroxyacyl-CoA dehydratase 2 (HACD2), 3-hydroxyacyl-CoA dehydratase 2 (HACD3), 3-hydroxyacyl-CoA dehydratase 4 (HACD4), hydroxysteroid 17-beta dehydrogenase 12 (HSD17B12), estradiol 17-beta-dehydrogenase 12-like (LOC508455 [37]), and trans-2-enoyl-CoA reductase (TECR). These interacting proteins are all lipid metabolism-related proteins. In terms of cellular component (CC), buffalo SCD is classified as an endoplasmic reticulum membrane protein (GO:0005789, GO:0016020, GO:0030176, https://www.ebi.ac.uk/QuickGO/, accessed on 28 June 2024). Its molecular functions (MFs) include stearoyl-CoA 9-desaturase activity (GO:0004768, https://www.ebi.ac.uk/QuickGO/, accessed on 28 June 2024), oxidoreductase activity acting on paired donors to reduce molecular oxygen to two water molecules (GO:0016717, https://www.ebi.ac.uk/QuickGO/, accessed on 28 June 2024), and metal ion binding (GO:0046872, https://www.ebi.ac.uk/QuickGO/, accessed on 28 June 2024). In addition, SCD is involved in the PPAR and AMPK signaling pathways.

### 3.7. Tissue Differential Expression

**Figure 7:**
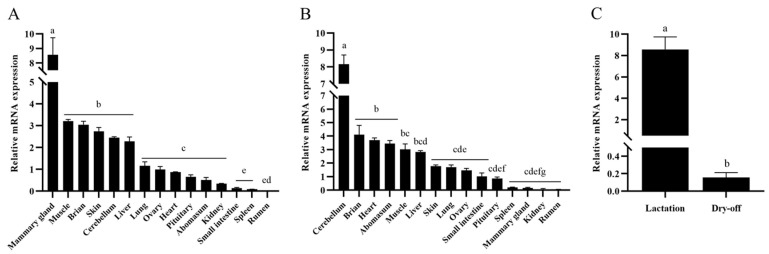
Differential expression of the *SCD* gene across multiple tissues in buffalo. (**A**) Differential expression of the *SCD* gene in 15 tissues during the lactation period; (**B**) differential expression of the *SCD* gene in 15 tissues during the dry-off period; (**C**) differential expression of the *SCD* gene in the mammary gland during lactation and dry-off periods. Different letters (a, b, c, d, e, f, g) indicate significant differences between groups (*p* < 0.05).

In the 15 tissues of lactating buffalo examined, the *SCD* gene expression was highest in the mammary gland, followed by the muscle, brain, skin, cerebellum, and liver, with the lowest expression in the small intestine, spleen, and rumen (Figure 7A). In the 15 tissues of dry-off period buffalo examined, the highest expression of the *SCD* gene was found in the cerebellum, followed by the brain, heart, abomasum, muscle, and liver, with the lowest expression in the spleen, mammary gland, kidney, and rumen (Figure 7B). Compared to other tissues, the *SCD* gene showed higher expression levels in the brain, muscle, and liver in both physiological periods, and lower expression levels in the spleen, kidney and rumen. It is noteworthy that the expression level of the *SCD* gene in the mammary gland of buffalo was significantly higher in lactation than in dry-off period (Figure 7C).

### 3.8. Subcellular Localization of the Buffalo SCD

**Figure 8:**
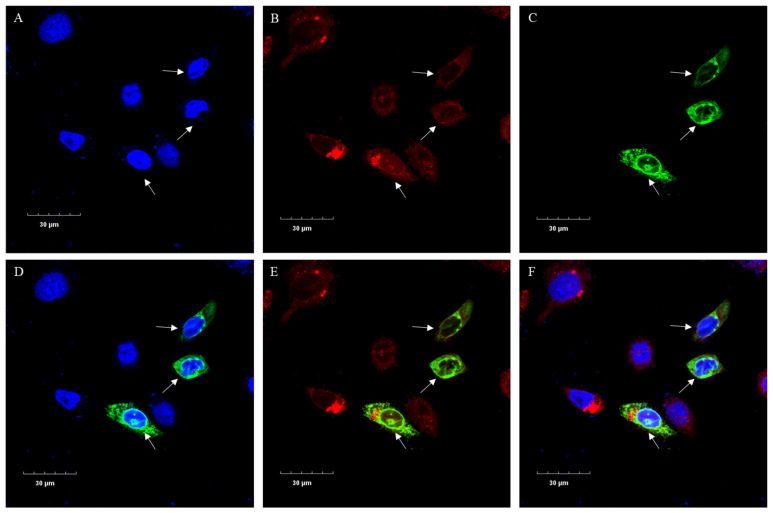
Subcellular localization of buffalo SCD in BuMECs observed using confocal microscopy. (**A**) Nuclei stained with Hoechst 33342; (**B**) mitochondria stained with Mito Tracker; (**C**) the green fluorescence of GFP encoded by recombinant pEGFP-N1-*SCD*; (**D**) the overlay of GFP and Hoechst-stained nuclei; (**E**) the overlay of GFP and Mito Tracker-stained mitochondria; (**F**) the combined overlay of GFP, nuclei, and mitochondria.

Subcellular localization predictions indicated that the buffalo SCD protein primarily functions in the endoplasmic reticulum membrane (score of 9.12), followed by the cell membrane (score of 0.83) and the nucleus (score of 0.05). To verify the distribution of SCD in BuMECs, we conducted subcellular localization experiments. After transfecting BuMECs with the constructed recombinant vector pEGFP-N1-*SCD*, the results showed that the green fluorescent protein (GFP) mainly overlapped with the red fluorescence of the mitochondria in the cytoplasm, while it did not overlap with the blue fluorescence of the nucleus (Figure 8), indicating that the buffalo SCD protein primarily functions in the cytoplasm.

### 3.9. Effects of SCD Overexpression on Lipid Synthesis-Related Genes in BuMECs

**Figure 9:**
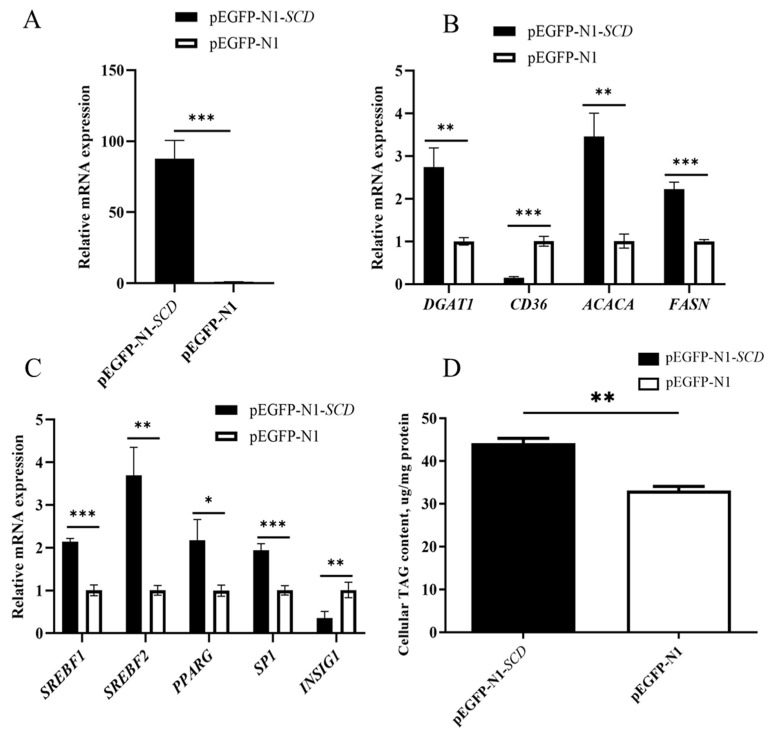
Effects of *SCD* overexpression on milk fat synthesis-related genes in BuMECs. (**A**) Differences in *SCD* expression in BuMECs after transfection with pEGFP-N1-*SCD* and pEGFP-N1; (**B**) expression differences in genes involved in de novo fatty acid synthesis (*ACACA*, *FASN*), fatty acid esterification (*DGAT1*), and fatty acid extracellular transport and uptake (*CD36*) following *SCD* overexpression; (**C**) changes in the expression of regulatory genes related to fatty acid synthesis (*SREBF1*, *SREBF2*, *PPARG*, *INSIG1*, *SP1*) due to *SCD* overexpression; (**D**) overexpression of *SCD* increased intracellular TAG content. Values are presented as means ± SEM; * *p* < 0.05, ** *p* < 0.01, *** *p* < 0.001.

The recombinant vector pEGFP-N1-*SCD* was transfected into the BuMECs, with pEGFP-N1 used as the negative control. qPCR results showed that the abundance of *SCD* mRNA in BuMECs increased significantly (~87-fold, *p* < 0.001) compared to the control group (Figure 9A). At the same time, *SCD* overexpression led to an increase in the mRNA abundance of *ACACA* (~3.43-fold), *FASN* (~2.22-fold), and diacylglycerol O-acyltransferase 1 (*DGAT1*) (~2.74-fold), which are involved in de novo fatty acid synthesis and fatty acid esterification. However, the mRNA abundance of *CD36*, which is involved in fatty acid uptake and transport, decreased by 85% (Figure 9B), indicating that *SCD* overexpression positively impacts the expression of genes related to de novo milk fat synthesis. Furthermore, the mRNA abundance of key regulatory genes in milk fat synthesis, including sterol regulatory element-binding transcription factor 1 (*SREBF1*) (~2.13-fold), sterol regulatory element-binding transcription factor 2 (*SREBF2*) (~3.68-fold), peroxisome proliferator-activated receptor gamma (*PPARG*) (~2.78-fold), and *SP1* transcription factor (~1.94-fold), also increased, while insulin-induced gene 1 (*INSIG1*) mRNA abundance significantly decreased by 65% (Figure 9C). This suggests that *SCD* overexpression affects the expression of core genes regulating milk fat synthesis. To determine the effect of *SCD* overexpression on TAG accumulation in the BuMECs, we measured the changes in TAG content. Accompanying *SCD* overexpression, the TAG levels in the BuMECs significantly increased by ~1.34-fold (*p* < 0.01) (Figure 9D).

### 3.10. Effects of SCD Knockdown on Lipid Synthesis-Related Genes in BuMECs

**Figure 10:**
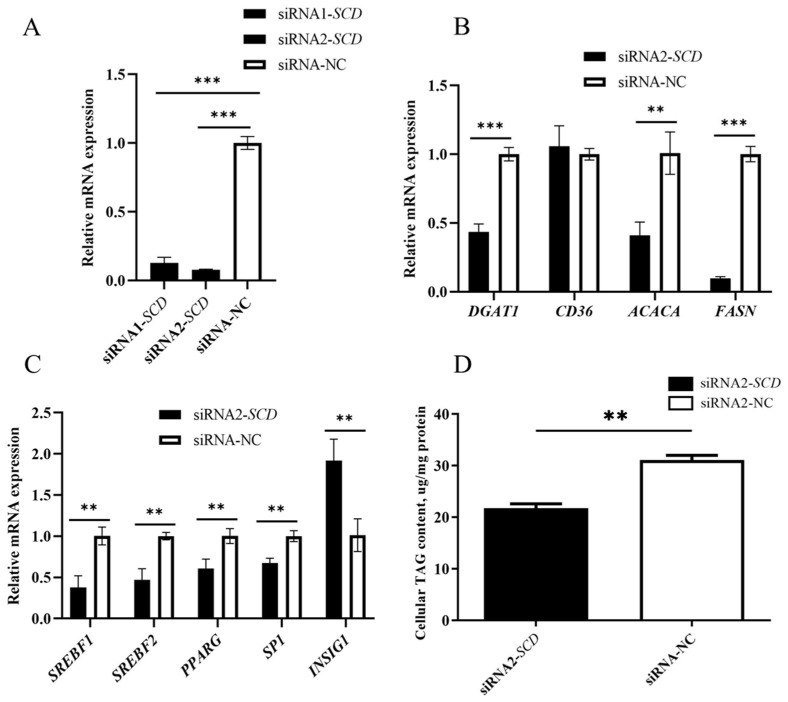
Effects of *SCD* knockdown on milk fat synthesis-related genes in BuMECs. (**A**) Interference efficiency of siRNA1-*SCD* and siRNA2-*SCD* compared to the negative control group (siRNA-NC); (**B**) effects of *SCD* knockdown on the expression of genes involved in fatty acid synthesis (*ACACA*, *FASN*), fatty acid esterification (*DGAT1*), and fatty acid extracellular transport and uptake (*CD36*); (**C**) effects of *SCD* knockdown on the expression of genes regulating milk fat metabolism (*SREBF1*, *SREBF2*, *PPARG*, *INSIG1*, *SP1*); (**D**) *SCD* knockdown reduced intracellular TAG content. Values are presented as means ± SEM.; ** *p* < 0.01; *** *p* < 0.001.

To assess the interference efficiency of two siRNAs targeting the *SCD* gene, siRNA-NC (negative control), siRNA1-*SCD*, and siRNA2-*SCD* were transfected into the BuMECs. The results showed that compared to the siRNA-NC control group, the mRNA abundance of *SCD* decreased by 87% after transfection with siRNA1-*SCD* (*p* < 0.001) and by 92% after transfection with siRNA2-*SCD* (*p* < 0.001) (Figure 10A). Since siRNA2-*SCD* exhibited higher interference efficiency, it was selected for subsequent cell transfection experiments.

siRNA2-*SCD* was transfected into the BuMECs to achieve *SCD* knockdown. The expression levels of genes related to milk fat synthesis were further analyzed following *SCD* knockdown. The mRNA abundance of *ACACA* and *FASN*, which are involved in de novo milk fat synthesis; *CD36*, which is involved in fatty acid uptake; *DGAT1*, which is associated with fatty acid esterification; and *SREBF1*, *SREBF2*, *PPARG*, and *SP1*, which regulate milk fat synthesis, decreased by 59%, 90%, 57%, 6%, 62%, 53%, 39%, and 33%, respectively, compared to the control group (*p* < 0.01 or *p* < 0.05). In contrast, the mRNA abundance of *INSIG1* increased by 1.89-fold (*p* < 0.01). Notably, *SCD* knockdown resulted in a 30% reduction in TAG content in the BuMECs (*p* < 0.01; Figure 10D).

### 3.11. Population Genetic Analysis and Association Analysis

A total of 16 SNPs were detected in the CDS of the *SCD* gene in both types of buffalo, and 2 SNPs were identified in the promoter region. Detailed results of the population genetic analysis are shown in Table 6. Among these SNPs, c.-603 (recorded with the first nucleotide of the start codon in the buffalo *SCD* gene-coding sequence as +1, NC_059179: g.21481146G>A), c.-605 (NC_059179: g.21481148A>C), c.609, c.867, and c.878 are shared between the two buffalo types. SNPs c.617, c.621, c.633, and c.987 are specific to river buffalo, while c.504, c.507, c.581, c.594, c.702, c.716, c.778, c.819, and c.842 are specific to swamp buffalo (Table 6). The SNPs c.581, c.617, c.716, c.778, c.842, and c.878 are nonsynonymous, resulting in amino acid changes p.Lys194Ile, p.Arg206Lys, p.Asp239Gly, p.Val260Ile, p.Asn281Ser, and p.Ala293Val, respectively. Analysis indicates that these nonsynonymous substitutions do not affect protein function. Additionally, in river buffalo, the SNPs at c.-605, c.609, and c.621 tend to be homozygous, while in swamp buffalo, all SNPs except at c.-603 and c.609 tend to be homozygous.

**Figure 11:**
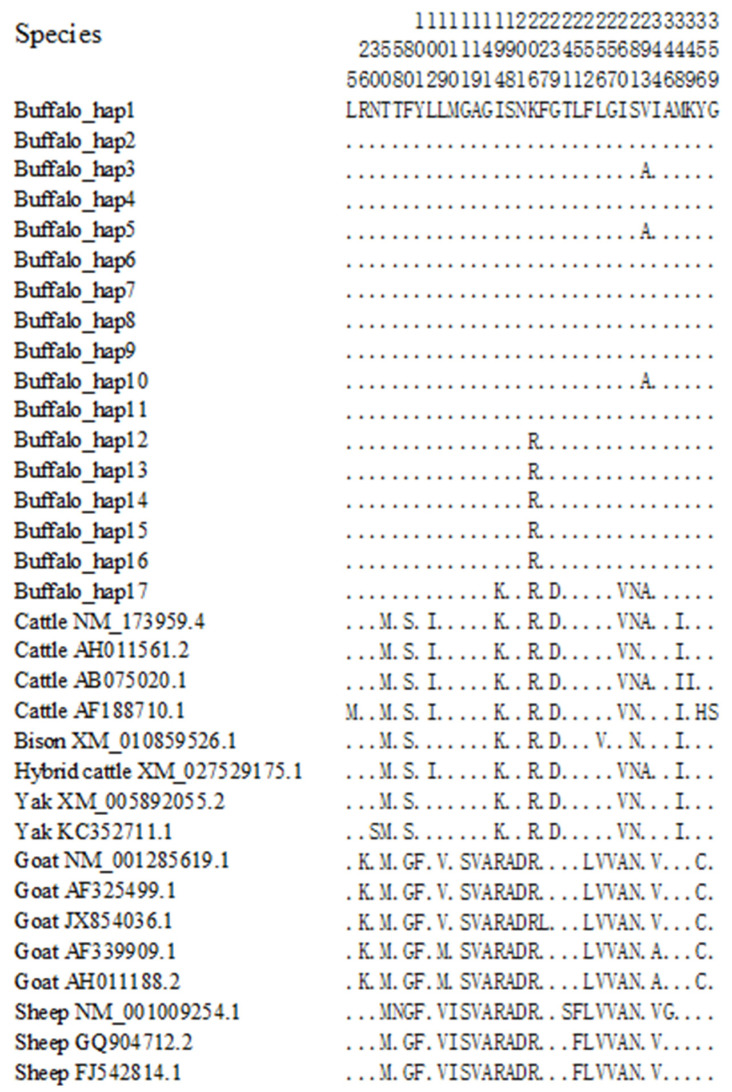
Differences in the SCD amino acid sequences between buffalo and other Bovidae species. The numbers represent the positions of the mature peptide. Dots (.) indicate identity with SCD, while amino acid substitutions are represented by different letters.

Based on the SNPs identified in the buffalo *SCD* CDS, a total of 17 haplotypes were defined, named Buffalo_hap1 through Buffalo_hap17 (Table 7). Among these, Buffalo_hap6 was the predominant haplotype, with a frequency of 0.2698, while Buffalo_hap14 had the lowest haplotype frequency, at 0.0032. To investigate the sequence variation characteristics of the buffalo *SCD* CDS, the buffalo haplotype sequences were compared with homologous sequences from other Bovidae species published in the NCBI database. The results revealed that c.108, c.149, and c.239 are nucleotide sites distinguishing buffalo from other Bovidae species (Figure S1), with c.149 and c.239 (p.50Thr and p.80Phe) also contributing to differences in the *SCD* amino acid sequences between buffalo and other Bovidae species (Figure 11).

Bioinformatics analysis confirmed that the six non-synonymous substitutions found in the CDS of buffalo *SCD* do not affect protein function and may not alter codon usage bias (Table S1). Therefore, this study focused solely on the association between variants in the *SCD* promoter region and buffalo lactation traits. The association analysis results (Table 8) indicate that in river buffalo, the AC genotype at the c.-605 site was associated with a significant 38.90% increase in milk yield compared to the AA genotype (*p* < 0.01), suggesting that the variation at this site has a potential impact on milk yield traits. Additionally, the genotype at the c.-603 site was not associated with buffalo lactation traits in this study.

## 4. Discussion

Milk, as a kind of human food with comprehensive nutrition, appropriate proportions, and ease of digestion and absorption, has seen increasing demand in recent years. SCD is a rate-limiting enzyme that catalyzes the conversion of SFAs (C16:0 and C18:0) into MUFAs (C16:1 and C18:1). Specifically, this reaction is aerobic, involving oxygen molecules, NAD(P)-cytochrome b5 reductase, and the electron acceptor cytochrome b5 [38]. The preferred substrates of SCD are palmitoyl-CoA and stearoyl-CoA, which are converted to palmitoleoyl-CoA and oleoyl-CoA, respectively [38]. MUFAs produced by SCD (such as phospholipids, TAG, cholesterol esters, wax esters, conjugated linoleic acid (CLA), and diacylglycerols) not only serve as key substrates for lipid synthesis [39] but also play important roles in signal transduction and cell differentiation (including neuronal differentiation) [40,41]. About 70% of the CLA in milk is synthesized by SCD, and CLA has anti-atherosclerotic, anti-cancer, and immune-regulatory functions [42]. From a human health perspective, the Wisconsin Milk Marketing Board (WMMB) suggests that the ideal fatty acid composition of milk should consist of 8% SFAs, 82% MUFAs, and 10% PUFAs [43]. This indicates that SCD plays a key role in the nutritional composition of milk and in maintaining human health. However, the role of the *SCD* gene during lactation in buffalo is still unclear.

In this study, the *SCD* gene was cloned and characterized from buffalo mammary tissue, with a CDS length of 1080 bp encoding 359 amino acids. The protein encoded by this gene plays a crucial role in fatty acid metabolism, particularly in the desaturation of fatty acids, and it also regulates cell membrane fluidity [44]. In terms of its molecular characteristics, such as transcript region structure, physicochemical properties, secondary structure, and tertiary structure, the buffalo SCD exhibits high similarity to that of other Bovidae species. Notably, phylogenetic analysis showed that buffalo clustered with other Bovidae, all of which share the conserved Delta9-FADS-like domain. This suggests that buffalo SCD is functionally similar to that of other Bovidae, i.e., SCD catalyzes the desaturation of fatty acids [45].

*SCD* is expressed and plays a functional role in organs associated with lipid metabolism in a variety of mammals. In mice, *SCD* is expressed in the liver, where it regulates the ratio of saturated to monounsaturated fatty acids as well as plasma TAG content [46]. In goats, *SCD* gene expression levels in mammary tissue are higher during lactation compared to dry-off periods [47,48]. In this study, we observed that *SCD* expression levels varied across 15 tissues in buffalo during lactation and dry-off periods, and the expression level of *SCD* in the mammary gland was higher during lactation than during dry-off, suggesting that *SCD* may be involved in the lactation process, particularly in milk fat synthesis in buffalo. Studies have shown that the endoplasmic reticulum (ER) is an important site for lipid metabolism [49], and Man et al. [50] found that *SCD* interacts with neighboring proteins on the ER in Hela cells, increasing the efficiency of TAG synthesis. Our subcellular localization and cellular function experiments in this study indicate that *SCD* functions within the ER of BuMECs.

De novo fatty acid synthesis is currently the main pathway for milk fat synthesis in cows [51]. Bionaz et al. [52] highlighted that SCD plays a pivotal role in de novo milk TAG synthesis in cows, which can be directly regulated by the transcription factors *SREBFs* and *PPAR*. Moreover, studies have shown that the AMP-activated protein kinase (*AMPK*)/mammalian target of rapamycin (*mTOR*) and phosphoinositide 3-kinase (*PI3K*)/protein kinase B (*PKB*) signaling pathways can enhance *SCD* expression [53,54]. In cow mammary epithelial cells, milk fat de novo synthesis is initially catalyzed by *ACACA* and *FASN*, which synthesize palmitic acid from acetyl-CoA and malonyl-CoA. Palmitic acid is then desaturated by *SCD* and catalyzed by *DGAT1* to form TAG [52,55]. Functional experiments in this study showed that overexpression or knockdown of the *SCD* gene in BuMECs led to corresponding changes in the mRNA abundance of genes (*ACACA* and *FASN*) involved in de novo milk fat synthesis, as well as in intracellular TAG content, indicating that the *SCD* gene is involved in de novo TAG synthesis in BuMECs. Palmitoyl-CoA is considered an inhibitor of *ACACA*, while palmitic acid is catalyzed to form TAG [56]. In this study, overexpression of *SCD* significantly increased TAG content in cells, suggesting that this process reduced palmitic acid levels, thereby alleviating the inhibitory effect of palmitoyl-CoA, ultimately leading to an increase in *ACACA* and *FASN* expression. This suggests a regulatory mechanism balancing lipid synthesis and breakdown in cells [57]. *CD36* promotes the preferential uptake of MUFAs in a *p38*- and *AMPK*-dependent manner, maintaining the balance between SFAs and MUFAs [58]. However, in this study, *SCD* overexpression was accompanied by a decrease in *CD36* expression, suggesting that overexpression of *SCD* may disrupt this balance.

SCD plays a crucial role in maintaining the balance of MUFAs. Even under MUFA-deficient dietary conditions, SCD can still synthesize MUFAs in the mouse liver [59]. The synthesis of sufficient amounts of MUFAs from scratch via dietary intake or SCD is closely associated with the activation of *SREBF1* and its downstream target genes *ACACA*, *FASN*, and *SCD*, and these activation processes further promote the synthesis of MUFAs and TAG [59]. In addition, *SCD* gene could not be activated by *SREBF1* after *SREBF1* was inhibited by *AMPK* [60]. In this study, we observed that *SREBF1* and *SREBF2* expression levels, as well as intracellular TAG content, were significantly altered following *SCD* overexpression or knockdown in BuMECs, suggesting that *SCD* indirectly affects the expression of *SREBFs*. In goat mammary epithelial cells, *PPARG* activation significantly increased *SCD* expression, while *PPARG* knockdown reduced *SCD* expression by 65% [61]. In this study, *SCD* overexpression or knockdown resulted in corresponding changes in *PPARG* expression and TAG content, indicating that *SCD* indirectly affects *PPARG* expression. Additionally, research has shown that chicken SP1 binds to the core promoter region of *SCD*, directly regulating *SCD* synthesis of polyunsaturated fatty acids [62]. This study found that *SCD* overexpression or knockdown also led to corresponding changes in *SP1* expression and intracellular TAG content, indicating that *SCD* is involved in lipid metabolism in BuMECs by indirectly affecting *SP1* expression. In summary, *SCD* indirectly influences *SREBFs*, *PPARG*, and *SP1* by catalyzing MUFA synthesis, activating downstream target genes related to lipid synthesis, and enhancing de novo TAG synthesis in BuMECs.

To date, SNPs in the *SCD* gene have been found to be closely associated with milk yield and milk fat composition in various Bovidae species [6,63]. In Holstein cows, the content of medium and long-chain unsaturated fatty acids (C14:1, C16:1, C18:2n6, and CLA) in the milk of individuals carrying the CT and TT genotypes was significantly higher than that of individuals carrying the CC genotype at the g.10329C>T locus of the *SCD* gene [64]. In cattle, buffalo, and goats, *SCD* has been identified as a candidate gene for lactation traits [65,66,67]. Gu et al. [68] found that the SNP at the g.133A>C locus in the promoter region of the river buffalo *SCD* gene was associated with its milk yield. In this study, a total of 18 SNPs were identified in the *SCD* gene of two types of buffalo, among which the SNPs at c.108, c.149, and c.239 could be used as specific markers distinguishing buffalo from other Bovidae species, while the SNP at the c.-605A>C locus resulted in significantly higher milk yield in individuals of Binglangjiang buffalo carrying the AC genotype than in individuals with the AA genotype. This finding suggests that the C allele at this locus is significantly associated with milk production in buffalo.

## 5. Conclusions

This study successfully cloned and characterized the *SCD* gene from buffalo, revealing its key role in fatty acid metabolism and milk fat synthesis. The expression of *SCD* in the mammary gland was higher during lactation than dry-off period, suggesting its involvement in milk fat synthesis. Functional experiments demonstrated that *SCD* influences the expression of key lipid synthesis genes, such as *ACACA* and *FASN*, and plays a role in regulating TAG content in BuMECs. This study also identified three SNPs (c.108, c.149, and c.239) in the *SCD* gene that could be used as specific loci to differentiate buffalo from other Bovidae species, and the SNP at the c.-605 site could be used as a marker to improve milk production traits in buffalo. These findings contribute to understanding the molecular mechanisms of milk fat synthesis in buffalo and may provide a basis for improving the productive performance of dairy buffalo through marker-assisted selection.

## Figures and Tables

**Table 1:** The information of the primers utilized in this study.

	Serial Number of Templates	Primer Sequence (5′→3′)	Product Length (bp)	Annealing Temperature (°C)	Extension Time (s)	Efficiency	Purpose
*SCD*	NM_001290915	F: TCAGGAACTAGTCTACACTCA	1292	59.70	65	/	Gene isolation
		R: GTACTGTAATGGGTTAACGT					
*SCD*	PP950448	F: ctcgagATGCCGGCCCACTTGCTGCA	1093	59.70	65	/	Construction of recombinant vector
		R: gaattcCGCCACTCTTGTAGCTTTCCT					
siRNA1-*SCD*	PP950448	F: GAAAAGAACUGGAGAGGAATT	/	/	/	/	Gene interference
		R: UUCCUCUCCAGUUCUUUUCTT					
siRNA2-*SCD*	PP950448	F: GGAGUCACCGAACCUACAATT	/	/	/	/	Gene interference
		R: UUGUAGGUUCGGUGACUCCTT					
siRNA-NC	/	F: UUCUCCGAACGUGUCACGUTT	/	/	/	/	Negative control
		R: ACGUGACACGUUCGGAGAATT					
*SCD*	NM_001290915	F: CGTGCCGTGGTATCTGTGG	217	60.00	20	2.10	Expression-level detection
		R: AAAGGTGTGGTGGTAGTTGTGG					
*SREBF2*	XM_025282779	F: GCCAAGATGCACAAGTCTGGTGTT	136	56.50	30	1.98	Expression-level detection
		R: TGCCCTTCAGGAGCTTGCTCT					
*CD36*	NM_001290838	F: CTTACAATAATACTGCAGATG	162	55.00	30	2.08	Expression-level detection
		R: AAGGTGGAAATGAGGCTG					
*SREBF1*	XM_025280442	F: GCACCGAGGCCAAGTTGAATAA	146	57.00	30	2.14	Expression-level detection
		R: CAGGTCCTTCAGTGATTTGCTT					
*SP1*	XM_025284254	F: CAGGATGGTTCAGGTCAGATAC	109	60.00	30	2.00	Expression-level detection
		R: GCTGGAGTAGGTTTGGCATAG					
*ACACA*	XM_025281124	F: CCTCTTCAGACAGGTTCAAGC	234	55.00	30	1.97	Expression-level detection
		R: TTCACCGCACACTGTTCCA					
*FASN*	XM_006061793	F: AGGCCAGCTCCGAAGGCAACA	209	64.30	30	2.01	Expression-level detection
		R: TACCACGTCGGCCACTTGTGTC					
*DGAT1*	NM_001290902	F: ACAGACAAGGACGGAGACG	268	55.00	30	2.04	Expression-level detection
		R: CCACAATGACCAGGCACA					
*PPARG*	NM_001290893	F: GCTCCAAGAGTACCAAAGTG	204	53.70	30	2.03	Expression-level detection
		R: GTCCTCCGGAAGAAACCCTT					
*INSIG1*	NM_001290924	F: ACGTTCAGCTCTCCTTGACATT	239	55.00	30	2.11	Expression-level detection
		R: CTGTCGTCCTATGTTTCCCAC					
*ACTB*	NM_001290932	F: TGGGCATGGAATCCTG	196	60.00	30	2.05	Internal reference
		R: GGCGCGATGATCTTGAT					
Exon 1–2	FN395259	F: TGGACTGCCCCGAACTCCG	1077	61.00	60	/	SNP detection
		R: TGCATCCCAACCCCCCTAG					
Exon 3	FN395259	F: CAAAGGAGCCTAAGAGAT	532	52.30	60	/	SNP detection
		R: AACAGAGGTTCAAAAATG					
Exon 4	FN395259	F: CTCACCACATAACCCTCG	761	54.10	60	/	SNP detection
		R: TCCTTCCACTCCCCAGTA					
Exon 5	FN395259	F: AGTGGAAAATCAGGTAGG	587	59.80	60	/	SNP detection
		R: TCAGAGATGACTGGGAAG					
Exon 6	FN395259	F: AGAGTTTAAAAGACGAGC	580	50.80	60	/	SNP detection
		R: TAATGGGAACAGAAGAGA					
Promoter	NC_059179	F: CCCAGTGCCCATCCATTTGC	557	53.30	60	/	SNP detection
		R: CGGACCCGACCTGCTGTGCT					

Note: Lowercase in the primer sequence indicates the enzyme cutting site (F: Xho1, R: EcoR1).

**Table 2:** Structural information on the *SCD* transcribed region of buffalo and other mammals.

Species	Accession Number	Length (bp)								
		5′UTR	E1	E2	E3	E4	E5	E6	CDS	3′UTR
Buffalo	NM_001290915.1	144	171	283	131	206	233	4060	1080	3863
Cattle	NM_173959.4	144	171	283	131	206	233	4073	1080	3876
Yak	XM_005892055.2	400	427	283	131	206	233	1963	1080	1766
Hybrid cattle	XM_027529175.1	432	459	283	131	206	233	4074	1080	3877
Bison	XM_010859526.1	395	422	283	131	206	233	4074	1080	3877
Goat	NM_001285619.1	0	27	283	131	206	233	200	1080	3
Sheep	NM_001009254.1	197	224	283	131	206	233	884	1080	687
Camel	XM_010974437.3	446	473	283	131	206	233	3967	1080	3767
Alpaca	XM_006210520.3	183	210	283	131	206	233	3965	1080	3768
Pig	NM_213781.1	176	203	283	131	206	233	4051	1080	3854
Rat	NM_139192.2	48	72	283	131	206	233	3550	1077	3353
Human	NM_005063.5	272	299	283	131	206	233	4093	1080	3896

**Table 3:** Physicochemical characteristics of SCD proteins in buffalo and other mammals.

Species	Number of Amino Acids	Molecular Weight (kDa)	Isoelectric Point (PI)	Instability Index (II)	Aliphatic Index (AI)	Negatively (Asp + Glu)/Positively (Arg + Lys) Charged Residues	Grand Average of Hydropathicity (GRAVY)
Buffalo_this study	359	41.70	9.23	47.21	87.24	32/41	–0.19
Cattle	359	41.71	9.22	43.99	86.43	33/42	–0.23
Yak	359	41.73	9.22	44.41	86.96	33/42	–0.23
Hybrid cattle	359	41.71	9.22	43.99	86.43	33/42	–0.23
Bison	359	41.73	9.22	45.64	86.96	33/42	–0.23
Goat	359	41.58	9.19	45.66	87.49	33/42	–0.18
Sheep	359	41.67	9.24	44.83	87.21	33/42	–0.20
Camel	359	41.51	9.23	43.82	87.49	32/42	–0.17
Alpaca	359	41.65	9.23	46.68	86.16	32/42	–0.18
Pig	359	41.38	9.23	47.35	89.16	32/42	–0.17
Rat	358	41.47	9.30	48.06	86.40	32/42	–0.24
Human	359	41.52	9.07	44.84	84.79	33/40	–0.19

**Table 4:** Functional modification sites of SCD proteins in buffalo and other Bovidae.

Functional Modification Sites	Serial No.	Location (AA) and Amino Composition
Casein kinase II phosphorylation site	PS00006	58–61: TyqD, 164–167: SetD, 166–169: TdaD, 309–312: SasE, 351–354: TgeE
Protein kinase C phosphorylation site	PS00005	95–97: TcK, 124–126: ShR, 127–129: TyK, 173–175: SrR, 355–357: SyK
N-myristoylation site	PS00008	85–90: GAlyGI, 114–119: GItaGA, 141–146: GNtmAF, 197–202: GstlNL, 257–262: GLnvTW
N-glycosylation site	PS00001	201–204: NLSD, 259–262: NVTW, 318–321: NFTT
cAMP- and cGMP-dependent protein kinase phosphorylation	PS00004	337–340: KKvS
Tyrosine kinase phosphorylation site 2	PS00007	349–356: KrtgEesY

**Table 5:** Secondary structure of SCD proteins in Bovidae species.

Structures	Buffalo_This Study	Cattle	Yak	Hybrid Cattle	Bison	Goat	Sheep
Random coil (%)	47.63	48.75	45.96	48.75	48.19	48.75	48.75
Alpha helix (%)	37.05	35.93	37.88	35.93	36.49	37.05	37.05
Beta turn (%)	4.74	4.46	5.01	4.46	5.01	3.34	3.34
Extended strand (%)	10.58	10.86	11.14	10.86	10.31	10.86	10.86

**Table 6:** Polymorphic sites of the *SCD* gene in two types of buffalo and their allele frequencies.

Population	SNPs	Genotype/Individual Number			Allele Frequency		Expected Heterozygosity	*p* Value ^1^
		WW	Wm	mm	W	m		
River buffalo	c.-603G>A	83	77	24	0.6604	0.3396	0.4528	0.3768
	c.-605A>C	167	17	0	0.9528	0.0472	0.0907	0.1030
	c.609C>T	178	6	0	0.9828	0.0172	0.0341	0.8940
	c.617G>A	142	36	6	0.8678	0.1322	0.2307	0.1450
	c.621C>T	171	11	2	0.9598	0.0402	0.0777	0.0089
	c.633G>A	80	80	24	0.6552	0.3448	0.4545	0.7148
	c.867A>C	129	17	38	0.7500	0.2500	0.3846	0.0005
	c.878T>C	36	17	131	0.2417	0.7583	0.3577	0.0001
	c.987C>A	17	74	93	0.3000	0.7000	0.4308	0.7405
Swamp buffalo	c.-603G>A	23	15	2	0.7625	0.2375	0.3668	0.0211
	c.-605A>C	36	4	0	0.9625	0.0375	0.0731	0.0400
	c.504T>C	38	0	2	0.9412	0.0588	0.0816	0.0000
	c.507T>C	38	0	2	0.9412	0.0588	0.0816	0.0000
	c.581T>A	38	0	2	0.9412	0.0588	0.0816	0.0000
	c.594T>C	38	0	2	0.9412	0.0588	0.0816	0.0000
	c.609C>T	31	7	2	0.8529	0.1471	0.2585	0.1488
	c.702G>A	38	0	2	0.9412	0.0588	0.0816	0.0000
	c.716G>A	38	0	2	0.9412	0.0588	0.0816	0.0000
	c.778A>G	38	0	2	0.9412	0.0588	0.0816	0.0000
	c.819T>C	38	0	2	0.9412	0.0588	0.0816	0.0000
	c.842G>A	38	0	2	0.9412	0.0588	0.0816	0.0000
	c.867A>C	38	0	2	0.9412	0.0588	0.0816	0.0000
	c.878T>C	38	0	2	0.9412	0.0588	0.0816	0.0000

^1^ The *p*-value of the Hardy–Weinberg equilibrium test.

**Table 7:** The haplotypes of the buffalo *SCD* gene and their frequencies.

Haplotype	Alleles	Actual Frequency	Expected Frequency
Buffalo_hap1	TTTTCACGGGATGATC	0.0549	0.0195
Buffalo_hap2	TTTTCACGGGATGATA	0.0797	0.0173
Buffalo_hap3	TTTTCACGGGATGACC	0.0201	0.0124
Buffalo_hap4	TTTTCACGGGATGCTC	0.0094	0.0064
Buffalo_hap5	TTTTCACGGGATGCCA	0.0060	0.0079
Buffalo_hap6	TTTTCACAGGATGATC	0.2698	0.0725
Buffalo_hap7	TTTTCACAGGATGATA	0.0671	0.0365
Buffalo_hap8	TTTTCACAGGATGCTC	0.0193	0.0195
Buffalo_hap9	TTTTCACAGGATGCTA	0.0115	0.0128
Buffalo_hap10	TTTTCACAGGATGCCC	0.1413	0.0621
Buffalo_hap11	TTTTCATAGGATGATC	0.0089	0.0074
Buffalo_hap12	TTTTCGCGGGATGATC	0.1960	0.0078
Buffalo_hap13	TTTTCGCGGGATGATA	0.0053	0.0053
Buffalo_hap14	TTTTCGCAGGATGATC	0.0032	0.0027
Buffalo_hap15	TTTTCGTGGGATGATC	0.0147	0.0035
Buffalo_hap16	TTTTTGCGGGATGATC	0.0378	0.0021
Buffalo_hap17	CCACCGCGAAGCACCC	0.0094	0.0005

**Table 8:** Association between SNP genotypes in the promoter region of the buffalo *SCD* gene and lactation traits.

Population	SNP	Genotype	Milk Yield (kg/d)	Milk Fat Rate (%)	Milk Protein Rate (%)	Lactose Rate (%)
River buffalo	c.-603G>A	GG (83)	5.4567 ± 0.3401	6.3540 ± 0.2311	4.2387 ± 0.0949	5.0844 ± 0.0556
		GA (76)	5.8084 ± 0.3785	6.7888 ± 0.2571	4.3900 ± 0.1056	5.0355 ± 0.0618
		AA (25)	5.5943 ± 0.6555	6.2468 ± 0.4453	4.2324 ± 0.1829	5.1578 ± 0.1071
	c.-605A>C	AA (166)	5.5179 ± 0.2462 ^A^	6.5361 ± 0.1734	4.2965 ± 0.0702	5.0827 ± 0.0409
		AC (18)	7.6644 ± 0.7629 ^B^	6.4773 ± 0.5373	4.0449 ± 0.2175	5.1626 ± 0.1266

Note: Different uppercase letters indicate a highly significant difference (*p* < 0.01).

## Data Availability

The data analyzed during the current study are available from the corresponding authors upon reasonable request.

## Supplementary Materials

The following supporting information can be downloaded at: https://www.mdpi.com/article/10.3390/ani14223191/s1, Figure S1: Differences between the haplotype sequences of the buffalo SCD CDS and homologous nucleotide sequences from other Bovidae species; Table S1: Sequence relative synonymous codon usage (RSCU) values after nonsynonymous substitution.
