# Supplementary material for: Molecular and Functional Analysis of the Stearoyl-CoA Desaturase (SCD) Gene in Buffalo: Implications for Milk Fat Synthesis
Source: Animals (Basel). 2024 Nov 7;14(22):3191. doi: 10.3390/ani14223191 (PMC11590957; doi:10.3390/ani14223191)
Supplement: Supplementary file 1 [file animals-14-03191-s001.zip › animals-3254139-supplementary.pdf]

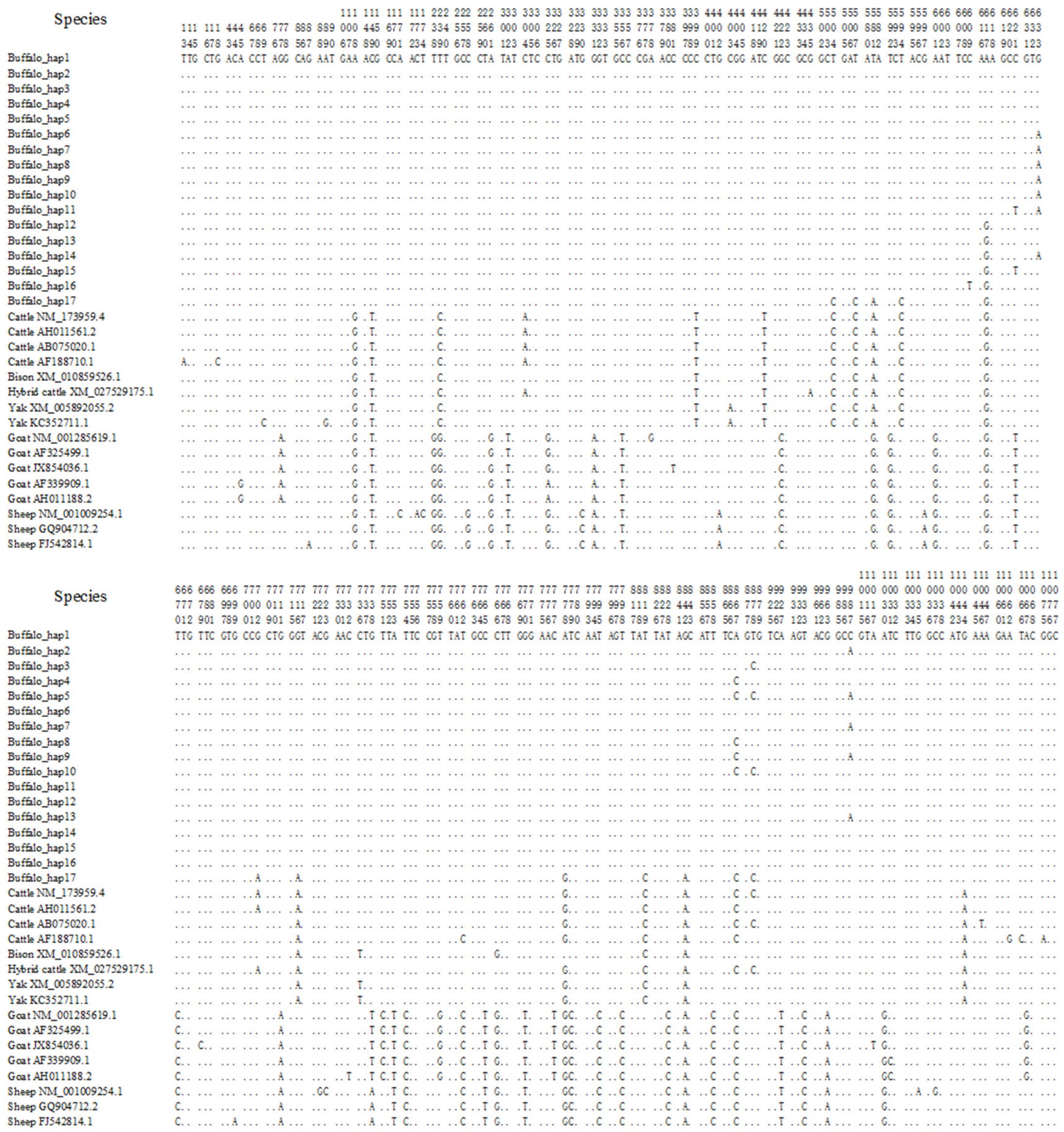

**Figure S1**. Differences between the haplotype sequences of the buffalo *SCD* CDS and homologous nucleotide sequences from other Bovidae species. The numbers represent nucleotide positions. Dots (.) indicate identity with the haplotype Buffalo_hap1, while nucleotide substitutions are represented by different letters.

**Table S1**. Sequence relative synonymous codon usage (RSCU) values after nonsynonymous substitution.

| Amino acids | Codons | Template sequence | c.581 | c.617 | c.716 | c.778 | c.842 | c.878 |
|---|---|---|---|---|---|---|---|---|
| Phe | UUU | 0.90 | 0.90 | 0.90 | 0.90 | 0.90 | 0.90 | 0.90 |
| | UUC | 1.10 | 1.10 | 1.10 | 1.10 | 1.10 | 1.10 | 1.10 |
| Leu | UUA | 0.57 | 0.57 | 0.57 | 0.57 | 0.57 | 0.57 | 0.57 |
| | UUG | 1.14 | 1.14 | 1.14 | 1.14 | 1.14 | 1.14 | 1.14 |
| | CUU | 0.43 | 0.43 | 0.43 | 0.43 | 0.43 | 0.43 | 0.43 |
| | CUC | 0.57 | 0.57 | 0.57 | 0.57 | 0.57 | 0.57 | 0.57 |
| | CUA | 0.57 | 0.57 | 0.57 | 0.57 | 0.57 | 0.57 | 0.57 |
| | CUG | 2.71 | 2.71 | 2.71 | 2.71 | 2.71 | 2.71 | 2.71 |
| Ile | AUU | 0.29 | 0.29 | 0.29 | 0.29 | 0.30 | 0.29 | 0.29 |
| | AUC | 2.43 | 2.43 | 2.43 | 2.43 | 2.40 | 2.43 | 2.43 |
| | AUA | 0.29 | 0.29 | 0.29 | 0.29 | 0.30 | 0.29 | 0.29 |
| Val | GUU | 0.86 | 0.86 | 0.86 | 0.86 | 0.80 | 0.86 | 0.80 |
| | GUC | 1.14 | 1.14 | 1.14 | 1.14 | 1.33 | 1.14 | 1.07 |
| | GUA | 0.57 | 0.57 | 0.57 | 0.57 | 0.53 | 0.57 | 0.53 |
| | GUG | 1.43 | 1.43 | 1.43 | 1.43 | 1.33 | 1.43 | 1.60 |
| Ser | UCU | 0.90 | 0.90 | 0.90 | 0.90 | 0.90 | 0.95 | 0.90 |
| | UCC | 2.10 | 2.10 | 2.10 | 2.10 | 2.10 | 2.21 | 2.10 |
| | UCA | 0.60 | 0.60 | 0.60 | 0.60 | 0.60 | 0.63 | 0.60 |
| | UCG | 0.00 | 0.00 | 0.00 | 0.00 | 0.00 | 0.00 | 0.00 |
| | AGU | 1.20 | 1.20 | 1.20 | 1.20 | 1.20 | 1.26 | 1.20 |
| | AGC | 1.20 | 1.20 | 1.20 | 1.20 | 1.20 | 0.95 | 1.20 |
| Pro | CCU | 1.33 | 1.33 | 1.33 | 1.33 | 1.33 | 1.33 | 1.33 |
| | CCC | 1.56 | 1.56 | 1.56 | 1.56 | 1.56 | 1.56 | 1.56 |
| | CCA | 0.67 | 0.67 | 0.67 | 0.67 | 0.67 | 0.67 | 0.67 |
| | CCG | 0.44 | 0.44 | 0.44 | 0.44 | 0.44 | 0.44 | 0.44 |
| Thr | ACU | 0.46 | 0.46 | 0.46 | 0.46 | 0.46 | 0.46 | 0.46 |
| | ACC | 2.00 | 2.00 | 2.00 | 2.00 | 2.00 | 2.00 | 2.00 |
| | ACA | 0.77 | 0.77 | 0.77 | 0.77 | 0.77 | 0.77 | 0.77 |
| | ACG | 0.77 | 0.77 | 0.77 | 0.77 | 0.77 | 0.77 | 0.77 |
| Ala | GCU | 1.28 | 1.12 | 1.28 | 1.28 | 1.28 | 1.28 | 1.33 |
| | GCC | 1.92 | 2.08 | 1.92 | 1.92 | 1.92 | 1.92 | 2.00 |
| | GCA | 0.48 | 0.48 | 0.48 | 0.48 | 0.48 | 0.48 | 0.50 |
| | GCG | 0.32 | 0.32 | 0.32 | 0.32 | 0.32 | 0.32 | 0.17 |
| Tyr | UAU | 1.00 | 1.00 | 1.00 | 1.00 | 1.00 | 1.00 | 1.00 |
| | UAC | 1.00 | 1.00 | 1.00 | 1.00 | 1.00 | 1.00 | 1.00 |
| His | CAU | 0.27 | 0.27 | 0.27 | 0.27 | 0.27 | 0.27 | 0.27 |
| | CAC | 1.73 | 1.73 | 1.73 | 1.73 | 1.73 | 1.73 | 1.73 |
| Gln | CAA | 0.67 | 0.67 | 0.67 | 0.67 | 0.67 | 0.67 | 0.67 |
| | CAG | 1.33 | 1.33 | 1.33 | 1.33 | 1.33 | 1.33 | 1.33 |
| Asn | AAU | 1.00 | 1.00 | 1.00 | 1.00 | 1.00 | 0.92 | 1.00 |
| | AAC | 1.00 | 1.00 | 1.00 | 1.00 | 1.00 | 1.08 | 1.00 |
| Lys | AAA | 0.74 | 0.74 | 0.67 | 0.74 | 0.74 | 0.74 | 0.74 |
| | AAG | 1.26 | 1.26 | 1.33 | 1.26 | 1.26 | 1.26 | 1.26 |
| Asp | GAU | 0.86 | 0.86 | 0.86 | 0.93 | 0.86 | 0.86 | 0.86 |
| | GAC | 1.14 | 1.14 | 1.14 | 1.07 | 1.14 | 1.14 | 1.14 |

| | | | | | | | | |
|---|---|---|---|---|---|---|---|---|
| Glu | GAA | 1.00 | 1.00 | 1.00 | 1.00 | 1.00 | 1.00 | 1.00 |
| | GAG | 1.00 | 1.00 | 1.00 | 1.00 | 1.00 | 1.00 | 1.00 |
| Cys | UGU | 0.00 | 0.00 | 0.00 | 0.00 | 0.00 | 0.00 | 0.00 |
| | UGC | 2.00 | 2.00 | 2.00 | 2.00 | 2.00 | 2.00 | 2.00 |
| Arg | CGU | 0.82 | 0.82 | 0.78 | 0.82 | 0.82 | 0.82 | 0.82 |
| | CGC | 1.36 | 1.36 | 1.30 | 1.36 | 1.36 | 1.36 | 1.36 |
| | CGA | 1.09 | 1.09 | 1.04 | 1.09 | 1.09 | 1.09 | 1.09 |
| | CGG | 0.82 | 0.82 | 0.78 | 0.82 | 0.82 | 0.82 | 0.82 |
| | AGA | 0.82 | 0.82 | 1.04 | 0.82 | 0.82 | 0.82 | 0.82 |
| | AGG | 1.09 | 1.09 | 1.04 | 1.09 | 1.09 | 1.09 | 1.09 |
| Gly | GGU | 1.22 | 1.22 | 1.22 | 1.09 | 1.22 | 1.22 | 1.22 |
| | GGC | 1.22 | 1.22 | 1.22 | 1.27 | 1.22 | 1.22 | 1.22 |
| | GGA | 0.87 | 0.87 | 0.87 | 0.91 | 0.87 | 0.87 | 0.87 |
| | GGG | 0.70 | 0.70 | 0.70 | 0.73 | 0.70 | 0.70 | 0.70 |
